# Unusual formation of (*E*)-11-(amino­methyl­ene)-8,9,10,11-tetra­hydro­pyrido[2′,3′:4,5]pyrimido[1,2-*a*]azepin-5(7*H*)-one and its crystal structure

**DOI:** 10.1107/S2056989017013093

**Published:** 2017-09-19

**Authors:** Khamid U. Khodjaniyazov, Utkir S. Makhmudov, Kambarali K. Turgunov, Burkhon Z. Elmuradov

**Affiliations:** a S.Yunusov Institute of the Chemistry of Plant Substances Academy of Sciences of, Uzbekistan Mirzo Ulugbek Str., 77, Tashkent 100170, Uzbekistan

**Keywords:** fused pyrimidines, formyl­ation, Vilsmeier–Haack reagent, X-ray structure analysis, crystal structure

## Abstract

Selective C-formyl­ation of 8,9,10,11-tetra­hydro­pyrido[2′,3′:4,5]pyrimido[1,2-*a*]-azepin-5(7*H*)-one has been studied for the first time. It was revealed that formyl­ation proceeds by the formation of an inter­mediate salt, which due to the re-amination process on treatment with aqueous ammonia transformed into the corresponding (*E*)-11-(amino­methyl­ene)-8,9,10,11-tetra­hydro­pyrido[2′,3′:4,5]-pyrimido[1,2-*a*]azepin-5(7*H*)-one, C_13_H_14_N_4_O, as an *E*-isomer. Formyl­ation was carried out by Vilsmeier–Haack reagent and the structure of the synthesized compound was confirmed by X-ray structural analysis, spectroscopic and LC–MS methods. In the mol­ecule, the seven-membered penta­methyl­ene ring adopts a twist-boat conformation.

## Abstract 2

Unusual formation of (*E*)-11-(amino­methyl­ene)-8,9,10,11-tetra­hydro-pyrido[2′,3′:4,5]pyrimido[1,2-*a*]azepin-5(7*H*)-one was found at formyl­ation of 8,9,10,11-tetra­hydro­pyrido[2′,3′:4,5]pyrimido[1,2-*a*]-azepin-5(7*H*)-one, which was explained by re-amination of firstly formed inter­mediate.

## Chemical context   

Pyrimidine-containing heterocyclic compounds are widely distributed in nature (Lagoja, 2005 ▸) and among synthetic compounds (Joshi *et al.*, 2016 ▸; Roopan & Sompalle, 2016 ▸). These compounds are of theoretical and practical inter­est, having plural reactivity and with many prospective biologically active compounds among the synthesized derivatives.

**Figure 1:**
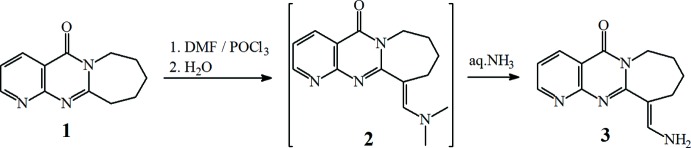
Reaction scheme.

In previous reports we have described several syntheses, *viz.* the reaction of 2,3-tri­methyl­enepyrido[2,3-*d*]pyrimidin-4-one with aromatic aldehydes (Khodjaniyazov, 2015*a* ▸,*b* ▸; Khodjaniyazov & Ashurov, 2016 ▸), selective reduction with sodium borohydride (Khodjaniyazov *et al.*, 2016*b* ▸), and the formation of (*E*)-9-(*N*,*N*-di­methyl­amino­methyl­idene)-8,9-di­hydro­pyrido[2,3-*d*]pyrrolo­[1,2-a]pyrimidin-5(7*H*)-one (Kho­djaniyazov *et al.*, 2016*a* ▸). In this current report we present the results of reaction of 8,9,10,11-tetra­hydro­pyrido[2′,3′:4,5]pyrimido[1,2-*a*]azepin-5(7*H*)-one (**1**) with the Vilsmeier–Haack reagent, decomposition by water and subsequent treatment with aqueous ammonia. We carried out the inter­action of **1** with a formyl­ating agent and, at the end of the reaction, the unusual final product (*E*)-11-(amino­methyl­ene)-8,9,10,11-tetra­hydro­pyrido[2′,3′:4,5]pyrimido-[1,2-*a*]azepin-5(7*H*)-one (**3**) was isolated after treatment (re-amination) of 11-di­methyl­amino­methyl­idene derivative (**2**) with aqueous ammonia. The reaction proceeds as shown in Fig. 1 ▸. The reaction product was different from that obtained in the case of formyl­ation of 2,3-tri­methyl­ene­pyrido[2,3-*d*]pyrimidin-4-one [pyrido[2,3-*d*]pyrrolo­[1,2-*a*]pyrimidin-5(7*H*)-one; Khodjaniyazov *et al.*, 2016*a* ▸]. This fact was explained by re-amination of the initially formed di­methyl­amino­methyl­idene derivative **2** under action of aqueous ammonia to give (*E*)-11-(amino­methyl­ene)-8,9,10,11-tetra­hydro­pyrido[2′,3′:4,5]pyrimido[1,2-*a*]azepin-5(7*H*)-one (**3**) as the final product
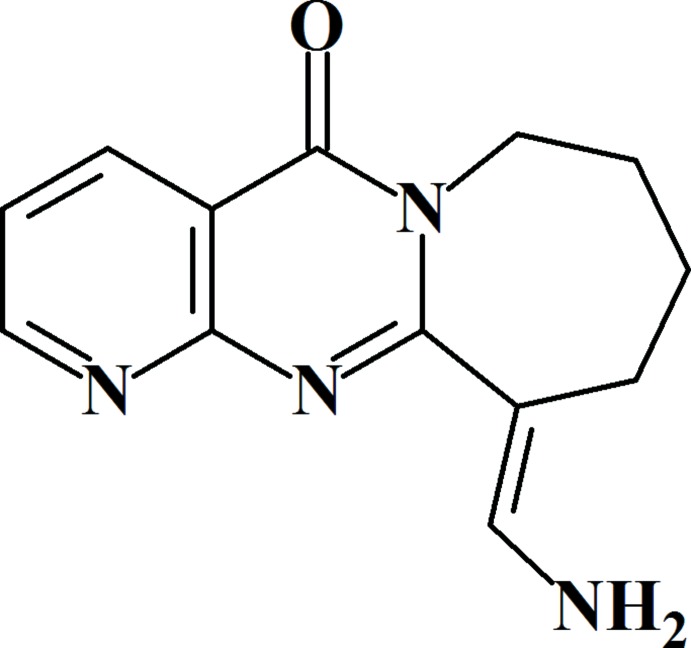
.

## Structural commentary   

**Figure 2:**
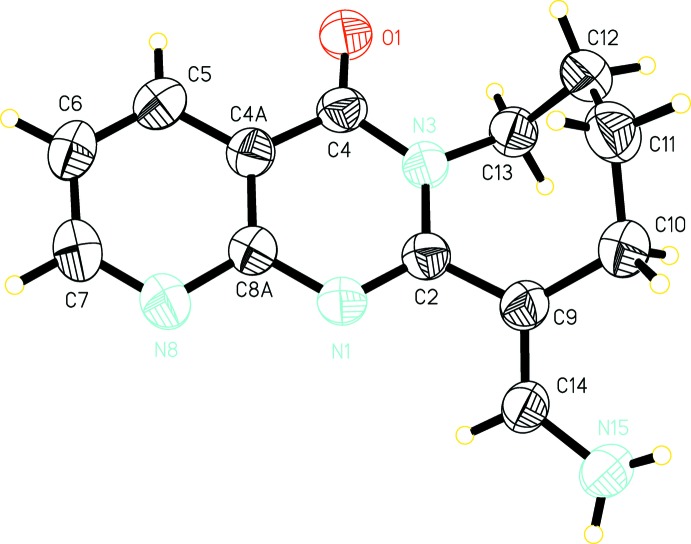
The mol­ecular structure of compound **3**, with the atom labelling and 50% probability displacement ellipsoids.

The title compound crystallizes in the centrosymmetric monoclinic *P2_1_/c* (No. 14) space group. The asymmetric unit contains one crystallographically independent mol­ecule. A displacement ellipsoid plot showing the atom-numbering scheme is presented in Fig. 2 ▸. In the mol­ecule, the seven-membered penta­methyl­ene ring exhibits a twist-boat conformation and has an approximate twofold symmetry with a *C_2_* axis passing through atom C12 and midpoint of the C2—C9 bond. The amino group is *E*-oriented and hybridization of the N atom in this group lies between *sp* ^3^ and *sp* ^2^. The C—N bond makes an angle of 155° with the bis­ector of the H—N—H angle. The equivalent angle in methyl­amine with a pyramidal *sp* ^3^-hybridized N atom is ∼123° (Klingebiel *et al.*, 2002 ▸) and it is nearly 180° in formamide with a planar sp^2^-hybridized N atom (Gajda & Katrusiak, 2011 ▸). The pyrimidine ring is twisted slightly, which may be because of the influence of the twisted seven-membered azepane ring. The N1—C8*A*—N4*A*—C4 torsion angle of is 8.7 (4)°.

## Supra­molecular features   

**Figure 3:**
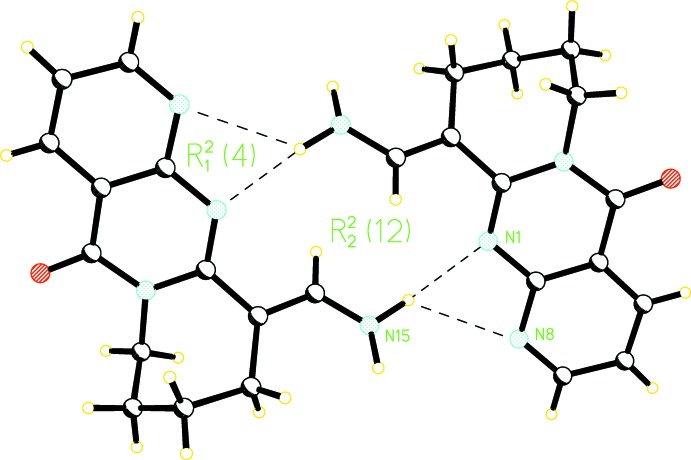
Hydrogen bonding in the title compound showing the 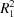
(4) and 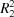
(12) graph-set motifs.

**Figure 4:**
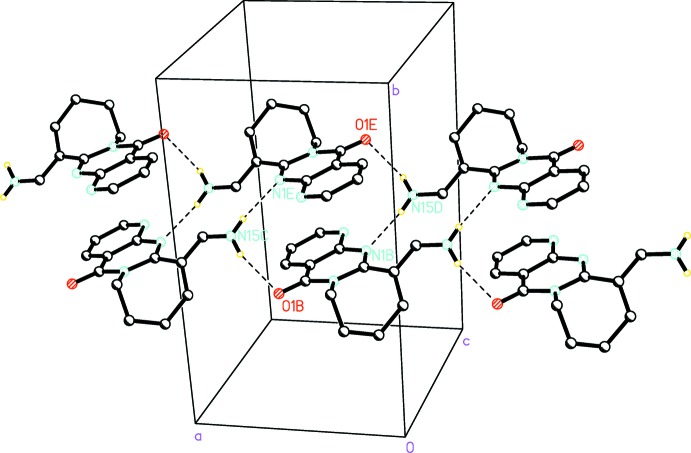
Hydrogen-bonded chain formation in **3**.

In the crystal, hydrogen bonds with 16 ring and three chain motifs are generated by N—H⋯N and N—H⋯O contacts (Table 1 ▸). The amino group is located close to the nitro­gen atoms N1 and N8 of an inversion-related mol­ecule, forming hydrogen bonds with 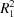
(4) and 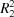
(12) graph-set motifs (Fig. 3 ▸). This amino group also forms a hydrogen bond with the C=O oxygen atom of a mol­ecule translated along the *a* axis, which links the mol­ecules into 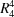
(16) rings. Hydrogen-bonded chains are formed along [100] by alternating 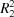
(12) and 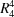
(16) rings (Fig. 4 ▸). These chains are stabilized by inter­molecular π-π-stacking inter­actions observed between the pyridine and pyrimidine rings [centroid–centroid distance = 3.669 (2) Å; symmetry operation 1 − *x*, 1 − *y*, 1 − *z*].

## Database survey   

A search of the Cambridge Structural Database (Version 5.38, last update November 2016; Groom *et al.*, 2016 ▸) for the 4-aza­quinazoline moiety gave eight hits. Only one of these is a related structure, a tricyclic 4-aza­quinazolin-4-one with a substituent on the third ring (VAMBET; Khodjaniyazov & Ashurov, 2016 ▸).

## Synthesis and crystallization   

**Materials and methods.** The results of electro spray ionization mass spectrometry (ESI–MS) were recorded using a 6420 TripleQuadLC/MC (Agilent Technologies, US) LC–MS spectrometer. The measurements were carried out in positive-ion mode. ^1^H NMR spectra were recorded in CD_3_OD on a Varian 400-MR spectrometer operating accordingly at 400 MHz. Hexa­methyl­disiloxcane (HMDSO) was used as inter­nal standard and the chemical shift of ^1^H was recorded in ppm. Melting points were measured on a Boetius and MEL–TEMP apparatus manufactured by Branstead inter­national (USA) and are uncorrected. IR spectra were recorded on an IR Fourier System 2000 (Perkin–Elmer) as KBr pellets.

The reaction process was monitored by TLC on Silufol UV-254 plates using a CHCl_3_/CH_3_OH (12:1) solvent system and the developed plates were visualized under a UV lamp. Solvents were purified by standard procedures. Organic solutions were dried over anhydrous Na_2_SO_4_ or with dried CaCl_2_.

**Synthesis of (** ***E*** **)-11-(amino­methyl­ene)-8,9,10,11-tetra­hydro­pyrido[2′,3′:4,5]-pyrimido[1,2-** ***a*** **]azepin-5(7** ***H*** **)-one** (**3**)**.** A round-bottom flask with freshly distilled DMF (3 ml, 39 mmol) was cooled by an ice–water bath and POCl_3_ (1 ml, 10.7 mmol) was added dropwise. The mixture was stirred (30 min), then 8,9,10,11-tetra­hydro­pyrido[2′,3′:4,5]pyrim­ido[1,2-*a*]azepin-5(7*H*)-one (**1**) (0.51 g, 2.4 mmol) was added into the reaction mixture. The reaction mixture was heated in a water bath for 1.5 h at 343 K and left for another day. Water (4 ml) was poured into the flask. TLC monitoring showed that the initial compound had fully transformed. The reaction mixture was treated by aqueous ammonia solution up to pH 9. The obtained solution was extracted by chloro­form (30 mL) three times. The chloro­form part was dried over Na_2_SO_4_ and the solvent was removed. Yield 0.34 g (60%), m.p. 458–460 K, *R*_f_ 0.63. Single crystals of **3** were grown from acetone solution by slow evaporation of the solvent at room temperature.

UV spectrum (ethanol, λ_max_, nm) neutral medium: 279.58, 348.97; acidic medium (HCl): 280.24, 362.37, 420.80; neutralization (HCl+NaOH): 279.12, 318.11, 362.29; basic medium (NaOH): 275.83, 347.71. IR spectrum (KBr, ν, cm^−1^): 3382 (NH_2_), 3325, 3203, 3064, 2924, 2869, 2824, 1642, 1613 (NH), 1591, 1562, 1523, 1470, 1433, 1389, 1353, 1319, 1267, 1249, 1227, 1184, 1126, 1107, 1077, 1045, 976, 934, 864, 825, 783, 735, 688, 663, 601, 548, 420. LC–MS (+ESI): 243 [*M*+H]^+^, 216.1, 201.1, 174, 160.9, 148.0, 121.0, 93.0, 79.0, 55.1, 39.1. ^1^H NMR spectrum [400 MHz, CD_3_OD, δ, ppm, *J* (Hz]): 1.77 (2H, *m*, γ-CH_2_), 1.92 (2H, *m*, δ-CH_2_), 2.385 (2H, *m*, β-CH_2_), 4.195 (2H, *t*, *J* = 6.1, ∊-CH_2_), 7.454 (1H, *br s*, =CH), 7.28 (1H, *dd*, *J* = 4.6, 7.9, H-6), 8.45 (1H, *dd*, *J* = 7.9, 2.1, H-5), 8.735 (1H, *dd*, *J* = 4.6, 2.1, H-7).

## Refinement   

Crystal data, data collection and structure refinement details are summarized in Table 2 ▸. H-bound N atoms were freely refined. C-bound H atoms were refined as riding with C—H = 0.93 or 0.97 Å and *U*_iso_(H) = 1.2*U*_eq_(C).

## Supplementary Material

Crystal structure: contains datablock(s) I, global. DOI: 10.1107/S2056989017013093/xu5905sup1.cif

Structure factors: contains datablock(s) I. DOI: 10.1107/S2056989017013093/xu5905Isup2.hkl

CCDC reference: 1530092

Additional supporting information:  crystallographic information; 3D view; checkCIF report

## Figures and Tables

**Table 1:** Hydrogen-bond geometry (Å, °)

*D*—H⋯*A*	*D*—H	H⋯*A*	*D*⋯*A*	*D*—H⋯*A*
N15—H1⋯O1^i^	0.85 (3)	2.21 (3)	3.017 (3)	159 (3)
N15—H2⋯N1^ii^	0.85 (5)	2.31 (5)	3.146 (3)	168 (4)
N15—H2⋯N8^ii^	0.85 (5)	2.79 (5)	3.401 (4)	131 (4)

Symmetry codes: (i) 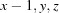; (ii) 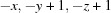.

**Table 2:** Experimental details

Crystal data	
Chemical formula	C_13_H_14_N_4_O
*M*_r_	242.28
Crystal system, space group	Monoclinic, *P*2_1_/*c*
Temperature (K)	293
*a*, *b*, *c* (Å)	8.7260 (7), 15.236 (3), 8.6642 (7)
β (°)	98.046 (8)
*V* (Å^3^)	1140.6 (3)
*Z*	4
Radiation type	Cu *K*α
μ (mm^−1^)	0.76
Crystal size (mm)	0.40 × 0.35 × 0.15

Data collection	
Diffractometer	Oxford Diffraction Xcalibur, Ruby
Absorption correction	Multi-scan (*CrysAlis PRO*; Oxford Diffraction, 2007 ▸)
*T*_min_, *T*_max_	0.965, 1.000
No. of measured, independent and observed [*I* > 2σ(*I*)] reflections	7589, 2328, 1478
*R*_int_	0.059
(sin θ/λ)_max_ (Å^−1^)	0.631

Refinement	
*R*[*F* ^2^ > 2σ(*F* ^2^)], *wR*(*F* ^2^), *S*	0.050, 0.141, 1.04
No. of reflections	2328
No. of parameters	171
H-atom treatment	H atoms treated by a mixture of independent and constrained refinement
Δρ_max_, Δρ_min_ (e Å^−3^)	0.18, −0.20

Computer programs: *CrysAlis PRO* (Oxford Diffraction, 2007 ▸), *SHELXS7* and *XP* in *SHELXTL* (Sheldrick, 2008 ▸) and *SHELXL2014/7* (Sheldrick, 2015 ▸).

## supplementary crystallographic information

### Crystal data

**Table:**

C_13_H_14_N_4_O	*D*_x_ = 1.411 Mg m^−^^3^
*M**_r_* = 242.28	Melting point: 458(2) K
Monoclinic, *P*2_1_/*c*	Cu *K*α radiation, λ = 1.54184 Å
*a* = 8.7260 (7) Å	Cell parameters from 911 reflections
*b* = 15.236 (3) Å	θ = 5.9–75.7°
*c* = 8.6642 (7) Å	µ = 0.76 mm^−^^1^
β = 98.046 (8)°	*T* = 293 K
*V* = 1140.6 (3) Å^3^	Plate, colourless
*Z* = 4	0.40 × 0.35 × 0.15 mm
*F*(000) = 512	

### Data collection

**Table:**

Oxford Diffraction Xcalibur, Ruby diffractometer	2328 independent reflections
Radiation source: Enhance (Cu) X-ray Source	1478 reflections with *I* > 2σ(*I*)
Graphite monochromator	*R*_int_ = 0.059
Detector resolution: 10.2576 pixels mm^-1^	θ_max_ = 76.7°, θ_min_ = 5.1°
ω scans	*h* = −9→10
Absorption correction: multi-scan (CrysAlis Pro; Oxford Diffraction, 2007)	*k* = −19→18
*T*_min_ = 0.965, *T*_max_ = 1.000	*l* = −10→8
7589 measured reflections	

### Refinement

**Table:**

Refinement on *F*^2^	0 restraints
Least-squares matrix: full	Hydrogen site location: mixed
*R*[*F*^2^ > 2σ(*F*^2^)] = 0.050	H atoms treated by a mixture of independent and constrained refinement
*wR*(*F*^2^) = 0.141	*w* = 1/[σ^2^(*F*_o_^2^) + (0.0554*P*)^2^ + 0.003*P*] where *P* = (*F*_o_^2^ + 2*F*_c_^2^)/3
*S* = 1.04	(Δ/σ)_max_ = 0.001
2328 reflections	Δρ_max_ = 0.18 e Å^−^^3^
171 parameters	Δρ_min_ = −0.20 e Å^−^^3^

### Special details

**Table:**

Geometry. All esds (except the esd in the dihedral angle between two l.s. planes) are estimated using the full covariance matrix. The cell esds are taken into account individually in the estimation of esds in distances, angles and torsion angles; correlations between esds in cell parameters are only used when they are defined by crystal symmetry. An approximate (isotropic) treatment of cell esds is used for estimating esds involving l.s. planes.

### Fractional atomic coordinates and isotropic or equivalent isotropic displacement parameters (Å^2^)

**Table:**

	*x*	*y*	*z*	*U*_iso_*/*U*_eq_	
O1	0.6098 (2)	0.35990 (15)	0.1710 (2)	0.0621 (5)	
N3	0.3638 (2)	0.39263 (13)	0.2102 (2)	0.0449 (4)	
N1	0.2921 (2)	0.40925 (14)	0.4621 (2)	0.0472 (5)	
N8	0.4731 (3)	0.39670 (16)	0.6821 (2)	0.0569 (5)	
N15	−0.1422 (3)	0.49351 (19)	0.2758 (3)	0.0606 (6)	
C2	0.2547 (3)	0.40679 (15)	0.3099 (3)	0.0438 (5)	
C9	0.0935 (3)	0.41797 (17)	0.2412 (3)	0.0478 (5)	
C4A	0.5549 (3)	0.36809 (16)	0.4323 (3)	0.0468 (5)	
C8A	0.4417 (3)	0.39271 (15)	0.5243 (3)	0.0459 (5)	
C4	0.5167 (3)	0.37221 (16)	0.2626 (3)	0.0476 (5)	
C14	0.0100 (3)	0.47631 (17)	0.3123 (3)	0.0491 (5)	
H14A	0.0630	0.5078	0.3950	0.059*	
C13	0.3195 (3)	0.40613 (17)	0.0415 (3)	0.0502 (6)	
H13A	0.4110	0.4200	−0.0056	0.060*	
H13B	0.2492	0.4556	0.0247	0.060*	
C5	0.7016 (3)	0.34430 (18)	0.5046 (3)	0.0557 (6)	
H5A	0.7782	0.3280	0.4457	0.067*	
C12	0.2420 (3)	0.32550 (18)	−0.0367 (3)	0.0560 (6)	
H12A	0.1995	0.3400	−0.1432	0.067*	
H12B	0.3189	0.2798	−0.0403	0.067*	
C6	0.7310 (3)	0.34538 (19)	0.6636 (3)	0.0576 (6)	
H6A	0.8268	0.3281	0.7154	0.069*	
C10	0.0156 (3)	0.3637 (2)	0.1043 (3)	0.0607 (7)	
H10A	−0.0772	0.3374	0.1341	0.073*	
H10B	−0.0163	0.4030	0.0177	0.073*	
C7	0.6134 (3)	0.3731 (2)	0.7466 (3)	0.0603 (6)	
H7A	0.6354	0.3750	0.8547	0.072*	
C11	0.1136 (3)	0.2911 (2)	0.0480 (3)	0.0617 (7)	
H11A	0.1589	0.2566	0.1368	0.074*	
H11B	0.0474	0.2527	−0.0214	0.074*	
H1	−0.201 (4)	0.456 (2)	0.224 (4)	0.112 (16)*	
H2	−0.181 (6)	0.527 (3)	0.338 (5)	0.16 (2)*	

### Atomic displacement parameters (Å^2^)

**Table:**

	*U*^11^	*U*^22^	*U*^33^	*U*^12^	*U*^13^	*U*^23^
O1	0.0464 (10)	0.0880 (14)	0.0538 (10)	0.0053 (9)	0.0141 (8)	−0.0095 (9)
N3	0.0398 (10)	0.0537 (11)	0.0415 (10)	−0.0006 (8)	0.0065 (8)	−0.0030 (8)
N1	0.0420 (10)	0.0574 (12)	0.0430 (10)	0.0022 (9)	0.0082 (8)	−0.0032 (8)
N8	0.0562 (13)	0.0707 (14)	0.0428 (11)	0.0026 (10)	0.0039 (9)	−0.0027 (9)
N15	0.0465 (13)	0.0786 (17)	0.0566 (13)	0.0100 (11)	0.0072 (10)	0.0016 (11)
C2	0.0416 (12)	0.0462 (12)	0.0440 (12)	−0.0003 (9)	0.0070 (9)	−0.0030 (9)
C9	0.0400 (12)	0.0593 (14)	0.0443 (12)	0.0014 (10)	0.0071 (9)	−0.0003 (10)
C4A	0.0424 (12)	0.0492 (13)	0.0484 (12)	−0.0002 (10)	0.0047 (10)	−0.0026 (9)
C8A	0.0430 (12)	0.0494 (13)	0.0450 (12)	0.0015 (9)	0.0049 (9)	−0.0019 (9)
C4	0.0404 (12)	0.0500 (12)	0.0533 (13)	0.0002 (10)	0.0097 (10)	−0.0076 (10)
C14	0.0393 (12)	0.0630 (15)	0.0446 (12)	0.0021 (11)	0.0042 (9)	0.0033 (10)
C13	0.0478 (13)	0.0601 (15)	0.0436 (12)	0.0002 (11)	0.0088 (10)	0.0018 (10)
C5	0.0450 (14)	0.0610 (16)	0.0611 (15)	0.0061 (11)	0.0074 (11)	−0.0003 (11)
C12	0.0616 (16)	0.0598 (16)	0.0457 (13)	0.0034 (12)	0.0044 (11)	−0.0082 (10)
C6	0.0467 (13)	0.0625 (16)	0.0600 (15)	0.0042 (11)	−0.0053 (11)	0.0051 (12)
C10	0.0461 (14)	0.0748 (18)	0.0598 (16)	−0.0004 (13)	0.0023 (11)	−0.0098 (13)
C7	0.0628 (16)	0.0693 (17)	0.0455 (13)	−0.0001 (13)	−0.0036 (11)	0.0008 (11)
C11	0.0616 (17)	0.0634 (17)	0.0589 (16)	−0.0091 (13)	0.0046 (12)	−0.0110 (12)

### Geometric parameters (Å, º)

**Table:**

O1—C4	1.227 (3)	C14—H14A	0.9300
N3—C4	1.383 (3)	C13—C12	1.516 (4)
N3—C2	1.389 (3)	C13—H13A	0.9700
N3—C13	1.473 (3)	C13—H13B	0.9700
N1—C2	1.314 (3)	C5—C6	1.365 (4)
N1—C8A	1.364 (3)	C5—H5A	0.9300
N8—C7	1.323 (4)	C12—C11	1.516 (4)
N8—C8A	1.357 (3)	C12—H12A	0.9700
N15—C14	1.347 (3)	C12—H12B	0.9700
N15—H1	0.852 (19)	C6—C7	1.398 (4)
N15—H2	0.849 (19)	C6—H6A	0.9300
C2—C9	1.458 (3)	C10—C11	1.519 (4)
C9—C14	1.352 (3)	C10—H10A	0.9700
C9—C10	1.525 (4)	C10—H10B	0.9700
C4A—C5	1.392 (3)	C7—H7A	0.9300
C4A—C8A	1.405 (3)	C11—H11A	0.9700
C4A—C4	1.463 (3)	C11—H11B	0.9700
			
C4—N3—C2	122.98 (19)	C12—C13—H13B	109.3
C4—N3—C13	117.70 (18)	H13A—C13—H13B	107.9
C2—N3—C13	119.16 (19)	C6—C5—C4A	118.8 (2)
C2—N1—C8A	118.76 (19)	C6—C5—H5A	120.6
C7—N8—C8A	117.3 (2)	C4A—C5—H5A	120.6
C14—N15—H1	120 (3)	C11—C12—C13	112.1 (2)
C14—N15—H2	116 (4)	C11—C12—H12A	109.2
H1—N15—H2	118 (4)	C13—C12—H12A	109.2
N1—C2—N3	122.2 (2)	C11—C12—H12B	109.2
N1—C2—C9	119.8 (2)	C13—C12—H12B	109.2
N3—C2—C9	118.0 (2)	H12A—C12—H12B	107.9
C14—C9—C2	116.2 (2)	C5—C6—C7	118.4 (2)
C14—C9—C10	120.1 (2)	C5—C6—H6A	120.8
C2—C9—C10	123.6 (2)	C7—C6—H6A	120.8
C5—C4A—C8A	119.3 (2)	C11—C10—C9	115.8 (2)
C5—C4A—C4	121.9 (2)	C11—C10—H10A	108.3
C8A—C4A—C4	118.7 (2)	C9—C10—H10A	108.3
N8—C8A—N1	115.9 (2)	C11—C10—H10B	108.3
N8—C8A—C4A	121.6 (2)	C9—C10—H10B	108.3
N1—C8A—C4A	122.3 (2)	H10A—C10—H10B	107.4
O1—C4—N3	121.1 (2)	N8—C7—C6	124.6 (2)
O1—C4—C4A	124.5 (2)	N8—C7—H7A	117.7
N3—C4—C4A	114.40 (19)	C6—C7—H7A	117.7
N15—C14—C9	126.7 (2)	C12—C11—C10	113.0 (2)
N15—C14—H14A	116.6	C12—C11—H11A	109.0
C9—C14—H14A	116.6	C10—C11—H11A	109.0
N3—C13—C12	111.7 (2)	C12—C11—H11B	109.0
N3—C13—H13A	109.3	C10—C11—H11B	109.0
C12—C13—H13A	109.3	H11A—C11—H11B	107.8
N3—C13—H13B	109.3		

### Hydrogen-bond geometry (Å, º)

**Table:**

*D*—H···*A*	*D*—H	H···*A*	*D*···*A*	*D*—H···*A*
N15—H1···O1^i^	0.85 (3)	2.21 (3)	3.017 (3)	159 (3)
N15—H2···N1^ii^	0.85 (5)	2.31 (5)	3.146 (3)	168 (4)
N15—H2···N8^ii^	0.85 (5)	2.79 (5)	3.401 (4)	131 (4)

Symmetry codes: (i) *x*−1, *y*, *z*; (ii) −*x*, −*y*+1, −*z*+1.
